# Identification and validation of biomarkers associated with mitochondrial dysfunction and ferroptosis in rat spinal cord injury

**DOI:** 10.3389/fneur.2025.1526966

**Published:** 2025-03-17

**Authors:** Jingliang Zhu, Shuai Wang, Yu Zhang, Chusong Zhou

**Affiliations:** ^1^Department of Orthopaedics, Zhujiang Hospital, Southern Medical University, Guangzhou, China; ^2^Department of Orthopaedics, General Hospital of Southern Theatre Command of PLA, Guangzhou, China

**Keywords:** spinal cord injury, ferroptosis, mitochondria, biomarker, machine learning

## Abstract

**Introduction:**

Mitochondrial dysfunction and ferroptosis have been implicated in the pathophysiological processes following spinal cord injury (SCI), with evidence suggesting their interplay influences neuronal cell survival and repair mechanisms. This study seeks to identify mitochondria- and ferroptosis-related biomarkers through comprehensive bioinformatics analysis.

**Methods:**

Mitochondria- and ferroptosis-associated differentially expressed genes (DEGs) were identified through the integration of differential expression analysis and weighted gene co-expression network analysis. Two machine learning algorithms, least absolute shrinkage and selection operator (LASSO) and Boruta, were employed to isolate SCI-associated feature genes. Biomarkers were subsequently identified by analyzing their expression levels. An artificial neural network (ANN) diagnostic model was constructed to predict SCI likelihood based on these biomarkers. Further evaluations were performed using enrichment analysis, immune infiltration profiling, molecular modulation assessment, and drug prediction. The biomarkers’ expression levels were validated using RT-qPCR.

**Results:**

In this study, two biomarkers, Hcrt and Cdca2, linked to mitochondrial function and ferroptosis in SCI, were found to be highly expressed in SCI samples. Tissue-specific analysis from the GTEx database revealed expression of these biomarkers in brain and spinal cord tissues. The ANN model, constructed using these biomarkers, accurately discriminated between SCI and control samples. Enrichment analysis highlighted several co-enriched pathways for Hcrt and Cdca2, including “ubiquitin-mediated proteolysis,” “endocytosis,” and the “neurotrophin signaling pathway.” Immune infiltration analysis, based on the Wilcoxon test, demonstrated significant differences in T follicular helper cell levels, which were lower in SCI samples compared to controls. Notably, T follicular helper cells exhibited a positive correlation with Hcrt and a negative correlation with Cdca2. Furthermore, seven transcription factors, including CEBPB, FOXC1, and GATA2, were identified as potential co-regulators of Hcrt and Cdca2. Drug prediction analysis revealed stable interactions of Cdca2 with pinosylvin, zinc acetate dihydrate, hydroquinone, lucanthone, and dasatinib. RT-qPCR validation confirmed the expression patterns of Hcrt and Cdca2 in alignment with the dataset, showing statistically significant differences.

**Discussion:**

This study identifies Hcrt and Cdca2 as biomarkers related to mitochondrial function and ferroptosis in SCI, providing new insights for the diagnosis and mechanistic understanding of SCI.

## Introduction

Spinal cord injury (SCI) represents a significant central nervous system disorder, typically resulting from external mechanical forces that compromise both the structural integrity and function of the spinal cord. Such disruption hinders motor, sensory, and reflex functions below the level of the lesion (1). Annually, 250,000 to 500,000 individuals worldwide experience traumatic spinal cord injuries, with this incidence expected to rise due to increased transportation usage and an aging population (2). Current clinical management of SCI predominantly involves surgical and pharmacological approaches (3). While these strategies may alleviate some symptoms, they remain insufficient in restoring neurological function. Consequently, there is a critical need for novel therapeutic strategies to address this pressing medical challenge.

Mitochondria are double-membraned organelles that play a crucial role in various physiological functions such as adenosine triphosphate (ATP) synthesis, calcium ion homeostasis, apoptosis, and reactive oxygen species (ROS) production regulation (4). In the context of SCI, mitochondrial dysfunction contributes significantly to secondary pathological alterations and neuronal death. Research on SCI models increasingly emphasizes mitochondrial impairment as a central factor affecting cellular metabolism, immune responses, axonal regeneration, and the renewal and differentiation of neural stem cells (5, 6). Ferroptosis, an iron-dependent form of regulated cell death, is triggered by the leakage of red blood cells, heme, and iron into injured tissues, leading to the generation of free radicals and subsequent toxicity (7). Ferroptosis plays a substantial role in SCI pathogenesis, with its acute phase occurring within 2 days and the subacute phase extending from 3 to 14 days post-injury (8). Furthermore, ferroptosis contributes to oxidative damage in neuronal cells following SCI by disrupting the redox balance (9). As primary ROS producers, mitochondria generate reactive species that accumulate, driving ferroptosis (10). Iron imbalance further aggravates mitochondrial dysfunction, impairing energy metabolism and increasing disease susceptibility (11). Beyond its role in iron uptake, storage, and utilization, mitochondrial iron overload aggravates dysfunction and ROS production, thereby promoting ferroptosis. However, the specific contributions of mitochondrial and ferroptosis-related genes in SCI pathogenesis remain incompletely understood. Further investigation into the interactions between mitochondria, ferroptosis, and SCI may yield insights for developing targeted therapeutic strategies, including novel drugs and treatment approaches for SCI.

This study identified mitochondrial and ferroptosis-related biomarkers through bioinformatics analysis of SCI-associated data from public databases. qPCR validation was performed on spinal cord samples from a rat SCI model collected on the second (acute phase) and seventh (subacute phase) days post-surgery, corresponding to the sample types in the database validation set. Additionally, molecular mechanisms of these biomarkers were examined using enrichment analysis, immune infiltration analysis, and regulatory network analysis, offering novel perspectives on SCI clinical treatment.

## Materials and methods

### Animal modeling

In SCI experimental models, urinary dysfunction is a common complication in animals. Female rats are typically selected for SCI studies owing to their relatively short and straight urethra, which simplifies postoperative management and minimizes the likelihood of urinary tract infections. For this study, 18 female SPF-grade SD rats, aged 8 weeks and weighing approximately 220 ± 20 grams, were sourced from Zhuhai Baishitong Biotechnology Co., Ltd. The animals were housed under standardized conditions, including controlled temperature, humidity, and a consistent light–dark cycle, with unrestricted access to food and water.

The rats were randomly assigned to three experimental groups: a sham surgery group (*n* = 6), a 2-day post-SCI group (*n* = 6), and a 7-day post-SCI group (*n* = 6). A SCI impact model was established to replicate human SCI. All procedures were performed at the Experimental Animal Research Center of Zhujiang Hospital, Southern Medical University (license number: SYXK (Guangdong) 2023-0215). The study received approval from the Ethics Committee of Zhujiang Hospital, Southern Medical University (approval number: LAEC-2024-120; approval date: June 21, 2024). Establishing the SCI model is a fundamental step in neuroscience research. In this study, a precise spinal cord impact device was employed to induce SCI in rats. Following anesthesia with isoflurane to ensure pain relief and unconsciousness, the rats were positioned prone, and the dorsal hair was shaved to prepare the area for surgery.

The surgical procedure involved a midline incision approximately 1.5 cm in length on the back. Using bony landmarks for guidance, the paraspinal muscles were dissected bluntly to fully expose the T10 vertebral lamina. A laminectomy was subsequently performed at the T10 level to facilitate the creation of the SCI model.

The study employed the 68099II spinal cord precision impactor from Shenzhen Ruiwoerd Life Science and Technology Co., Ltd., which offers precise control over impact speed, depth, and dwell time, ensuring accurate SCI induction. The impactor settings included a size 2 impact head, an impact velocity of 2 m/s, a depth of 1.2 mm, and a dwell time of 0.5 s. This controlled impact generated a significant hematoma at the targeted spinal segment.

Following SCI, the hematoma underwent dynamic changes over time. During the hyperacute phase, bleeding and inflammation exacerbated the hematoma, which gradually resolved in the later stages. This progression was typical of the natural course after SCI. The contusion also induced temporary involuntary spasms in the hind limbs and tail rigidity, serving as indicators of a successfully established and standardized SCI model.

Postoperatively, the muscles and skin were sutured in layers. To support the animals’ physiological function, manual bladder expression was performed three times daily for 1 week following the injury to ensure adequate urine elimination. In the sham group, rats underwent a laminectomy at the T10 level, preserving the spinal cord and maintaining normal BBB (Basso, Beattie, and Bresnahan) locomotor scores postoperatively.

At 2 and 7 days post-SCI, animals were anesthetized with a 40 mg/kg intraperitoneal injection of 1% pentobarbital sodium. Following cardiac perfusion with phosphate-buffered saline, spinal cord samples were harvested for subsequent qPCR analysis.

### Data acquisition

The gene expression omnibus (GEO) database^1^ provided SCI-associated microarray expression profiles, specifically GSE109902 and GSE166009. The GSE109902 dataset, derived from the GPL22396 platform, included 20 spinal cord tissue samples from SCI rats and 10 from control rats. The GSE166009 dataset, based on the GPL27597 platform, contained 12 spinal cord tissue samples, consisting of nine from SCI rats and three from controls. Additionally, 1,136 mitochondria-related human genes were obtained from the MitoCarta 3.0 database^2^ and subsequently mapped to rat homologs, resulting in 1,119 rat homologous genes (MRGs) (Supplementary Table S1). A total of 431 ferroptosis-related human genes retrieved from the literature were similarly converted to their rat homologs, yielding 437 rat genes (FRGs) (Supplementary Table S2) (12).

### Weighted gene co-expression network analysis

Expression data of MRGs and FRGs from the GSE109902 dataset were used to calculate single-sample gene set enrichment analysis (ssGSEA) scores via the GSVA package (v 1.42.0) (13). The Wilcoxon test was then applied to compare MRG and FRG scores between SCI and control groups (*p* < 0.05). A co-expression network was constructed using MRG and FRG scores as traits within the GSE109902 dataset through the weighted gene co-expression network analysis (WGCNA) package (v 1.7.1) (14). Outlier samples were first removed using hierarchical clustering based on Euclidean distance. The scale-free network evaluation coefficient (*R*^2^) and mean connectivity were subsequently used to determine the optimal soft-threshold power (*β*). Genes exhibiting similar expression patterns were grouped into the same module using the hybrid dynamic tree cutting algorithm, with parameters set as minModuleSize = 20, deepSplit = 2, and mergeCutHeight = 0.15. Different colors were assigned to modules for visualization. A heatmap of trait-module correlations was generated, emphasizing modules significantly associated with both MRG and FRG scores (|cor| >0.3 and *p* < 0.05). Genes within these modules were identified as key module genes, strongly correlated with MRG and FRG scores.

### Differential expression analysis and functional enrichment

Differentially expressed genes (DEGs) between SCI and control samples in the GSE109902 dataset were identified using the DESeq2 package (1.34.0) (15), with thresholds set at |log2 Fold Change (FC)| >0.5 and *p* < 0.05. A comprehensive visualization of DEG distribution was achieved by generating a volcano plot and heat map through the ggplot2 package (v 3.4.1) (16) and ComplexHeatmap-package (v 2.14.0) (17). A Venn diagram, constructed with the VennDiagram package (v 1.7.1) (18) was used to identify overlapping genes between key modular genes and DEGs, which were then classified as candidate genes.

These candidate genes were subjected to Gene Ontology (GO) annotation and Kyoto Encyclopedia of Genes and Genomes (KEGG) pathway enrichment analysis via the clusterProfiler package (v 4.2.2) (19), with pathways exhibiting *p* < 0.05 considered statistically significant. The co-expression network of candidate genes was constructed by mapping the genes onto GeneMANIA.^3^

### Machine learning algorithms and tissue-specific analysis

Two machine learning algorithms, least absolute shrinkage and selection operator (LASSO) and Boruta, were applied to identify feature genes associated with SCI from the candidate genes in the GSE109902 dataset. For LASSO analysis, the glmnet package (v 4.1–4) (20) was employed, with a lambda value of 0 selected as optimal for feature gene selection. Boruta analysis was performed using the Boruta package (v 8.0.0) (21) with default parameters. Subsequently, the common feature genes identified by both algorithms were determined through overlapping the results using the VennDiagram package (v 1.7.1).

Expression trends of the common feature genes were further compared between SCI and control samples in the GSE109902 and GSE166009 datasets using the Wilcoxon test. Emphasis was placed on genes exhibiting stable expression, defined as those with consistent expression patterns across both datasets and significant differential expression between groups (*p* < 0.05), which were selected as biomarkers for this study.

To assess tissue specificity, gene expression levels were analyzed using the online tool genotype-tissue expression (GTEx, https://gtexportal.org/home/).

### Construction of artificial neural network

An artificial neural network (ANN) diagnostic model was developed to predict SCI likelihood based on the expression levels of identified biomarkers. After normalizing data from the GSE109902 dataset, the model was constructed using the neuralnet package (v 1.44.2) (22), employing the min-max method and configuring three hidden layers. To evaluate the model’s predictive accuracy, the receiver operating characteristic (ROC) curve was generated using the pROC package (v 1.18.0) (23). An area under the curve (AUC) exceeding 0.7 was considered indicative of high accuracy.

### Enrichment analysis of biomarkers

To elucidate the signaling pathways related to the biomarkers, GSEA was conducted on the GSE109902 dataset. Initially, Spearman correlation analysis was performed between the biomarkers and other genes across all samples using the psych package (v 2.1.6). Genes were then ranked based on their correlation coefficients, from highest to lowest. Concurrently, GSEA was carried out using the clusterProfiler package (v 4.2.2), with the “c2.cp.kegg.v7.4.symbols.gmt” gene set from the Molecular Signatures Database (MSigDB, http://www.gsea-msigdb.org/gsea/msigdb/index.jsp) as the reference. The thresholds for statistical significance were set at adj. *p* < 0.05 and |Normalized Enrichment Score (NES)| >1.

Additionally, the biomarkers were analyzed using the GeneMANIA database (see text footnote 3) to predict genes associated with the biomarkers’ functions and the biological processes in which they participate.

### Immune infiltration analysis

Immune cell infiltration plays a critical role in characterizing disease defense mechanisms, offering insights into the extent of immune involvement in specific pathologies. In this study, infiltration levels of 22 immune cell types were quantified in samples from the GSE109902 dataset using the CIBERSORT algorithm (24). Differential immune cell infiltration between SCI and control samples was then assessed via the Wilcoxon test (*p* < 0.05). Additionally, Spearman correlation coefficients were calculated to examine the relationships between the immune cell types, as well as between biomarkers and immune cell infiltration, based on the expression matrix of biomarkers and immune cell levels.

### Correlation analysis with inflammatory factors

The SCI process is typically associated with the release of inflammatory factors (25). To explore the relationship between biomarkers and inflammatory factors, 200 inflammation-related genes (IRGs) were retrieved from the MSigDB database using the search term “HALLMARK_INFLAMMATORY_RESPONSE.” These 200 IRGs formed the background gene set for subsequent analysis. ssGSEA was then conducted on the GSE109902 dataset via the GSVA package to calculate the IRG score for each sample. Differences in IRG scores between the SCI and control groups were assessed (*p* < 0.05). Additionally, the relationship between biomarkers and IRGs was examined by calculating Spearman correlation coefficients and their significance, with the thresholds set at |cor| >0.3 and *p* < 0.05.

### Molecular regulatory network analysis

To investigate the regulatory factors targeting the biomarkers, upstream transcription factors (TFs) were identified through the miRNet database.^4^ Additionally, microRNAs (miRNAs) regulating the biomarkers were detected using miRecords,^5^ miRTarBase,^6^ and TarBase.^7^ The upstream long non-coding RNAs (lncRNAs) of the identified miRNAs were further explored in the same databases to elucidate the molecular mechanisms governing the biomarkers. Finally, the TF-biomarker and lncRNA-miRNA-mRNA (biomarker) networks were visualized using Cytoscape software (v 3.10.1) (26) based on the predicted results.

### Drug prediction and molecular docking

Potential drugs targeting biomarkers were identified through an in-depth analysis of the Enrichr database^8^ to support drug prediction and inform therapeutic strategies for SCI. The relationship between biomarkers and drugs was further examined by obtaining biomarker protein structures from the Protein Data Bank (PDB) database.^9^ Molecular docking was conducted using the Chemical and Biological Docking (CB-Dock) platform,^10^ focusing on interactions with binding energies below 5 kcal/mol, indicative of stronger biomarker-drug affinity. The docking results were subsequently visualized using PyMOL software (v 3.0.3) (27) to facilitate structural interpretation and analysis.

### Real-time quantitative polymerase chain reaction

Spinal cord samples from T10 were collected at 2 and 7 days post-SCI, immediately frozen, and stored at −80°C (six samples per group). Total RNA was extracted using TRIzol reagent (Thermo Fisher Scientific, Inc.) and reverse transcribed into complementary DNA (cDNA). Thermal cycling was performed under the following conditions: denaturation at 95°C for 30 s, annealing at 95°C for 3 s, and extension at 60°C for 30 s. GAPDH mRNA served as the endogenous control, and results were quantified using the $2^{-\mathrm{\Delta \Delta CT}}$ method. The primers utilized in this study were listed in Table 1. Since the experiment involved comparing data from postoperative day 2 and postoperative day 7 with the control group, we selected one-way analysis of variance (one-way ANOVA) as the statistical analysis method.

**Table 1:** Primers of RT-qPCR used in this study.

Gene	Primers forward (5′–3′)	Product size (bp)
HCRT	Forward: TTC TAC AAA GGT TCC CTG GG	20
	Reverse: AAC AGT TCG TAG AGA CGG C	19
CDCA2	Forward: TCT CCA CAG TAA CCG TAG AG	20
	Reverse: GGG AAG ATG ATG ACT TTC CTG	21
GAPDH	Forward: ACT CTA CCC ACG GCA AGT TC	20
	Reverse: TGG GTT TCC CGT TGA TGA CC	20

### Statistical analysis

Data processing and statistical analysis were conducted using R software (v 4.2.2). Group differences were assessed using the Wilcoxon test, with statistical significance set at *p* < 0.05.

## Results

### A total of 3,098 key module genes were obtained through WGCNA

**Figure 1:**
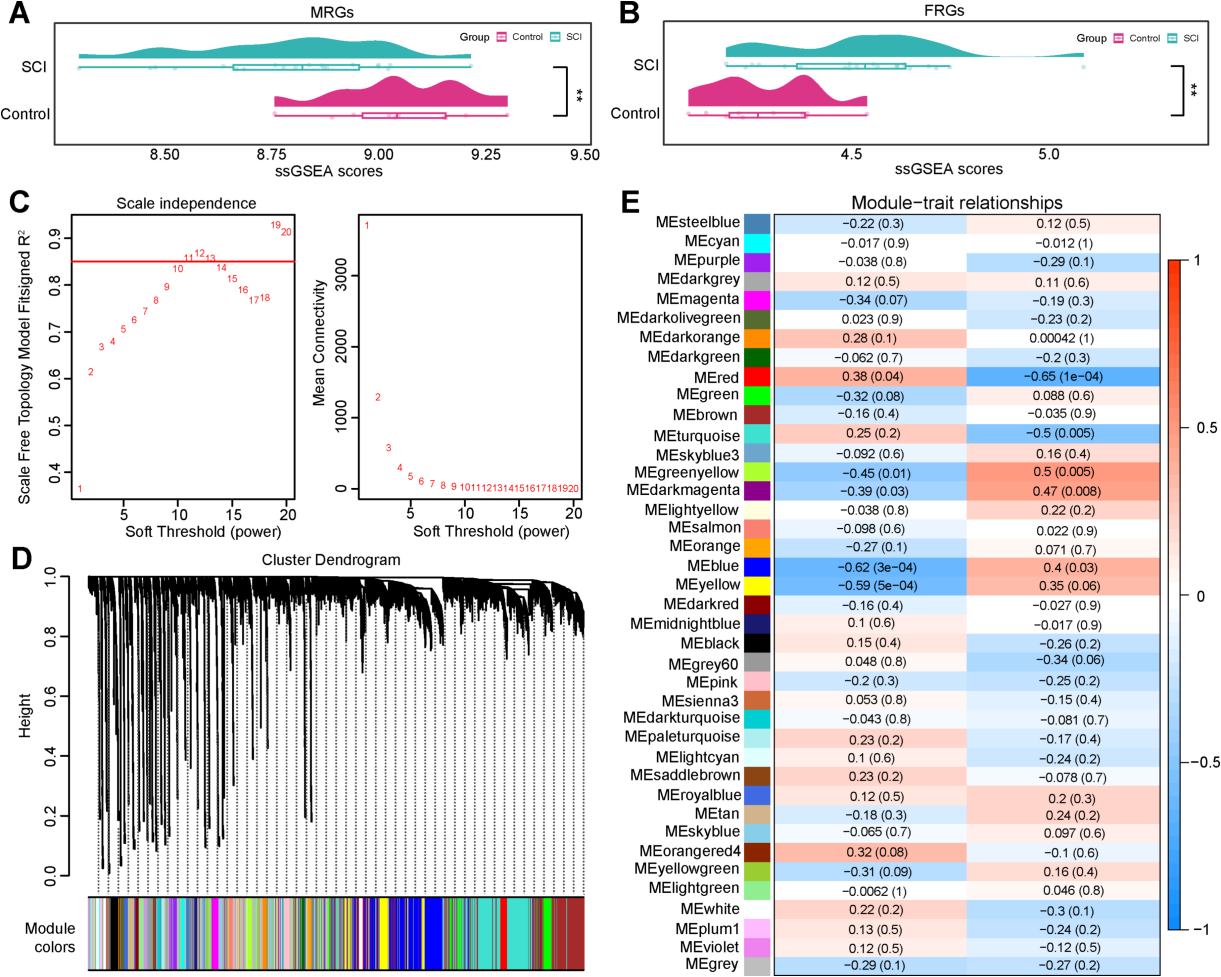
WGCNA analyses of MRGs and FRGs. **(A,B)** A comparison of ssGSEA scores for MRGs and FRGs. **(C)** Optimal threshold screening curve for network modules. **(D)** Gene-module clustered tree diagram. **(E)** Correlation heat map of the module with MRGs and FRGs.

The expression matrices of MRGs and FRGs in the GSE109902 dataset revealed significant differences in both the MRGs and FRGs scores between the SCI and control groups, with the SCI group exhibiting a lower MRGs score and a higher FRGs score (*p* < 0.05) (Figures 1A,B), as determined by the ssGSEA algorithm. WGCNA was subsequently employed to identify gene clusters strongly associated with the MRGs and FRGs scores. Cluster analysis revealed no outlier samples. When the *β*-value was set to 11, the *R*^2^ surpassed 0.85 (red line) and the mean connectivity approached zero, indicating optimal network consistency with a scale-free distribution and biological relevance (Figure 1C). Hierarchical clustering analysis identified 39 co-expression modules (excluding gray modules) (Figure 1D). Using a threshold of |cor| >0.3 and *p* < 0.05, four modules—MEblue, MEred, MEgreenyellow, and MEdarkmagenta—were found to be significantly correlated with the MRGs and FRGs scores (Figure 1E). In total, 3,098 genes within these modules were identified as key module genes associated with both the MRGs and FRGs scores.

### Elucidation of biological functions associated with eight candidate genes

**Figure 2:**
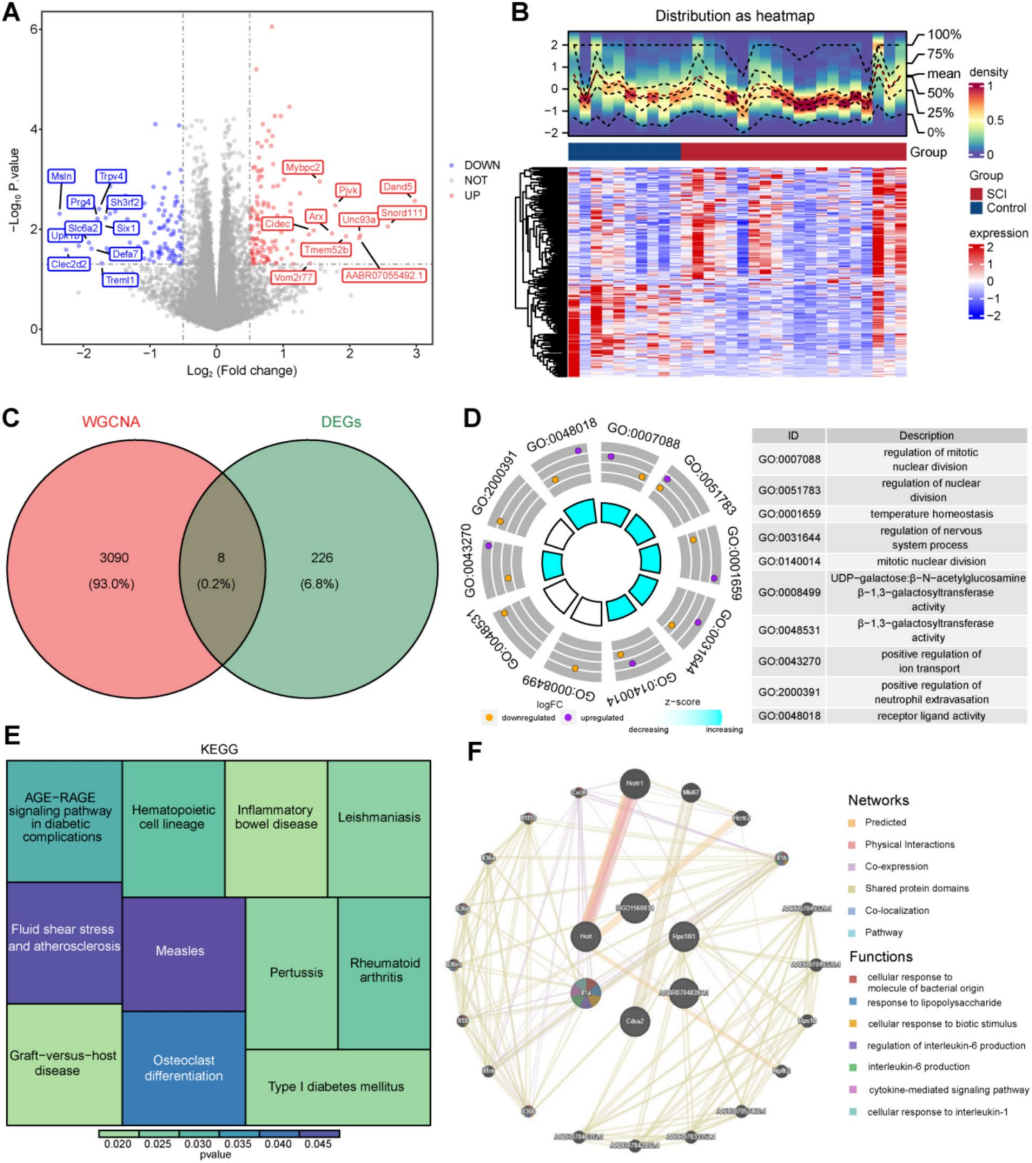
Identification of candidate genes and GO and KEGG enrichment analysis and GeneMANIA network construction. **(A,B)** Diagram of DEGs volcanoes and heat map. **(C)** Venn diagram of candidate genes. **(D,E)** GO and KEGG enrichment analysis. **(F)** GeneMANIA analysis of prognostic genes.

Using the criteria of |log_2_FC| >0.5 and *p* < 0.05, 234 DEGs, consisting of 129 upregulated and 105 downregulated genes, were identified between SCI and control samples in GSE109902 (Figures 2A,B). Overlapping 3,098 key modular genes with the DEGs revealed eight mitochondrial and ferroptosis-associated genes, designated as candidate genes: U4, AABR07048397.1, Hcrt, Rps18l1, Cdca2, SNORD115, RGD1560034, and Il1a (Figure 2C).

These candidate genes underwent enrichment analysis, resulting in 301 GO terms, including 280 biological processes (BPs), two cellular components (CCs), and 19 molecular functions (MFs), as well as 11 KEGG pathways (*p* < 0.05). The top 10 GO terms were predominantly related to “regulation of mitotic nuclear division,” “regulation of nervous system processes,” “temperature homeostasis,” “positive regulation of ion transport,” and “positive regulation of neutrophil extravasation,” among others (Figure 2D). KEGG analysis highlighted the involvement of candidate genes in “inflammatory bowel disease,” “graft-versus-host disease,” and “osteoclast differentiation,” among other pathways (Figure 2E).

Furthermore, a GeneMANIA database search identified 20 functionally similar genes (e.g., Hertrl, Mki67, Hertr2), and a co-expression network was constructed to elucidate their roles in various processes, including “regulation of interleukin 6 production,” “interleukin 6 production,” and “cytokine-mediated signaling pathways” (Figure 2F).

### Hcrt and Cdca2 were determined to be biomarkers in SCI

**Figure 3:**
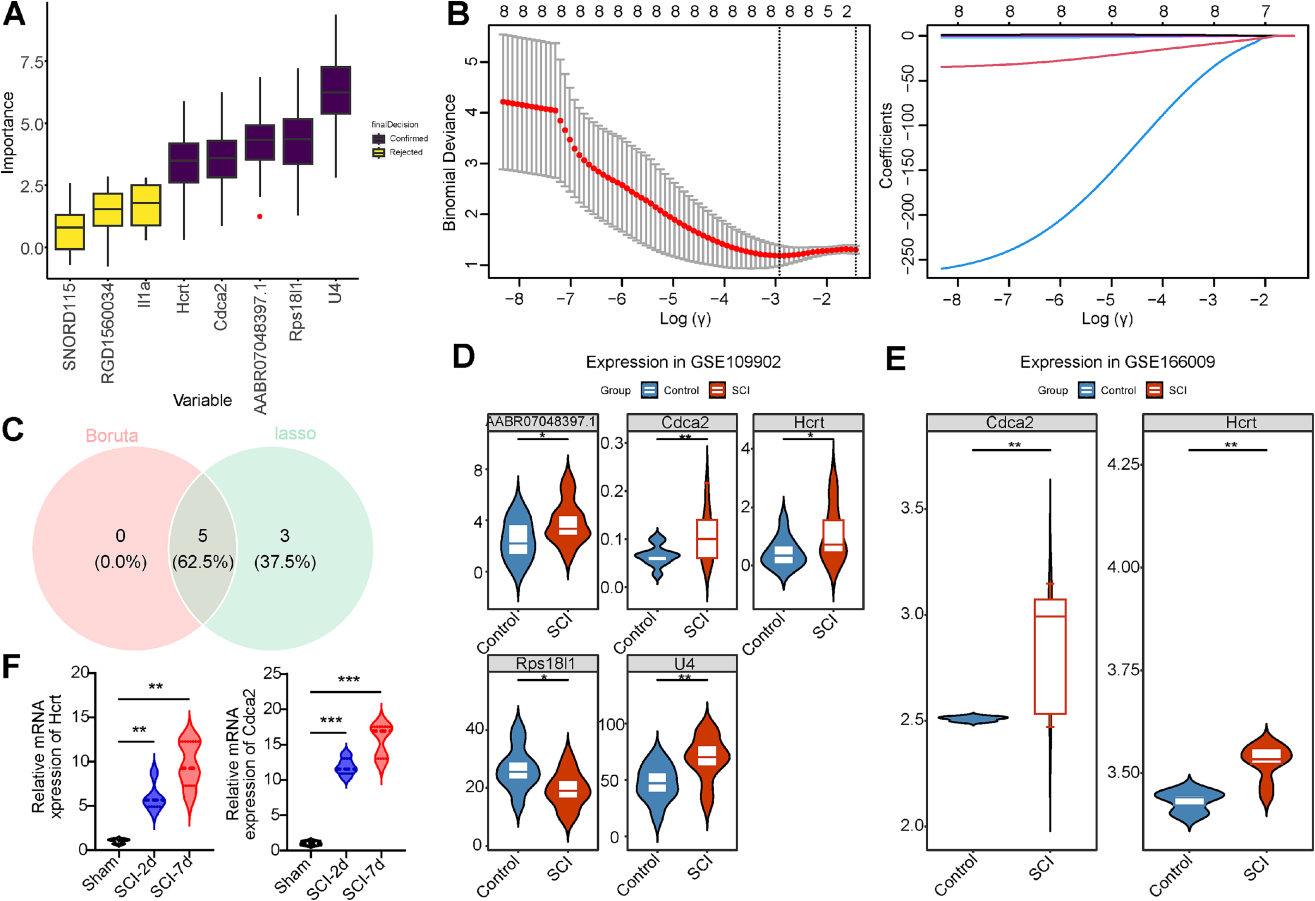
Hcrt and Cdca2 were determined to be biomarkers in SCI. **(A)** Boruta’s algorithm identifies candidate trait genes. **(B)** Cross-validation of LASSO regression analyses and spectrogram of LASSO coefficients. **(C)** Feature genes Venn diagram, with green representing the feature genes identified by lasso and red representing the feature genes identified by Boruta. **(D)** Expression profiles of candidate biomarker genes in GSE109902 (*p* < 0.05). **(E)** Expression status of candidate biomarker genes in GSE166009 (*p* < 0.05). **(F)** qPCR results of Hcrt and Cdca2 genes in the injured spinal cord and normal samples (^*^p < 0.05, ^**^p < 0.01, and ^***^p < 0.001).

Two machine learning algorithms were employed to identify the most significant feature genes associated with SCI from the eight candidate genes. The Boruta algorithm highlighted five key genes (U4, AABR07048397.1, Hcrt, Rps18l1, Cdca2) based on feature importance (Figure 3A). In LASSO analysis, conducted at a lambda_min_ value of 0.05360385, eight feature genes were selected: U4, AABR07048397.1, SNORD115, RGD1560034, Hcrt, Rps18l1, Il1a, and Cdca2 (Figure 3B). An overlap analysis between the two algorithms revealed five common feature genes: U4, AABR07048397.1, Hcrt, Rps18l1, and Cdca2 (Figure 3C).

The expression level analysis revealed significant differences in the five genes between the SCI and control groups in the GSE109902 dataset (*p* < 0.05) (Figure 3D). Similarly, Hcrt and Cdca2 exhibited significant differential expression between groups in the GSE166009 dataset (*p* < 0.05) (Figure 3E). qPCR validation was performed on rat spinal cord tissue samples collected at days 2 and 7 post-SCI surgery, showing a marked increase in the expression of both Hcrt and Cdca2 following the procedure. These results align with the expression patterns observed in the GSE166009 validation dataset, confirming the reliability of the screening process (Figure 3F). Notably, both Hcrt and Cdca2 demonstrated elevated expression in SCI samples compared to control samples across the two datasets. However, no expression of U4, AABR07048397.1, or Rps18l1 was detected in the GSE166009 dataset. Consequently, Hcrt and Cdca2 were selected as biomarkers for subsequent analyses.

### Biomarkers specifically expressed in brain—spinal cord

**Figure 4:**
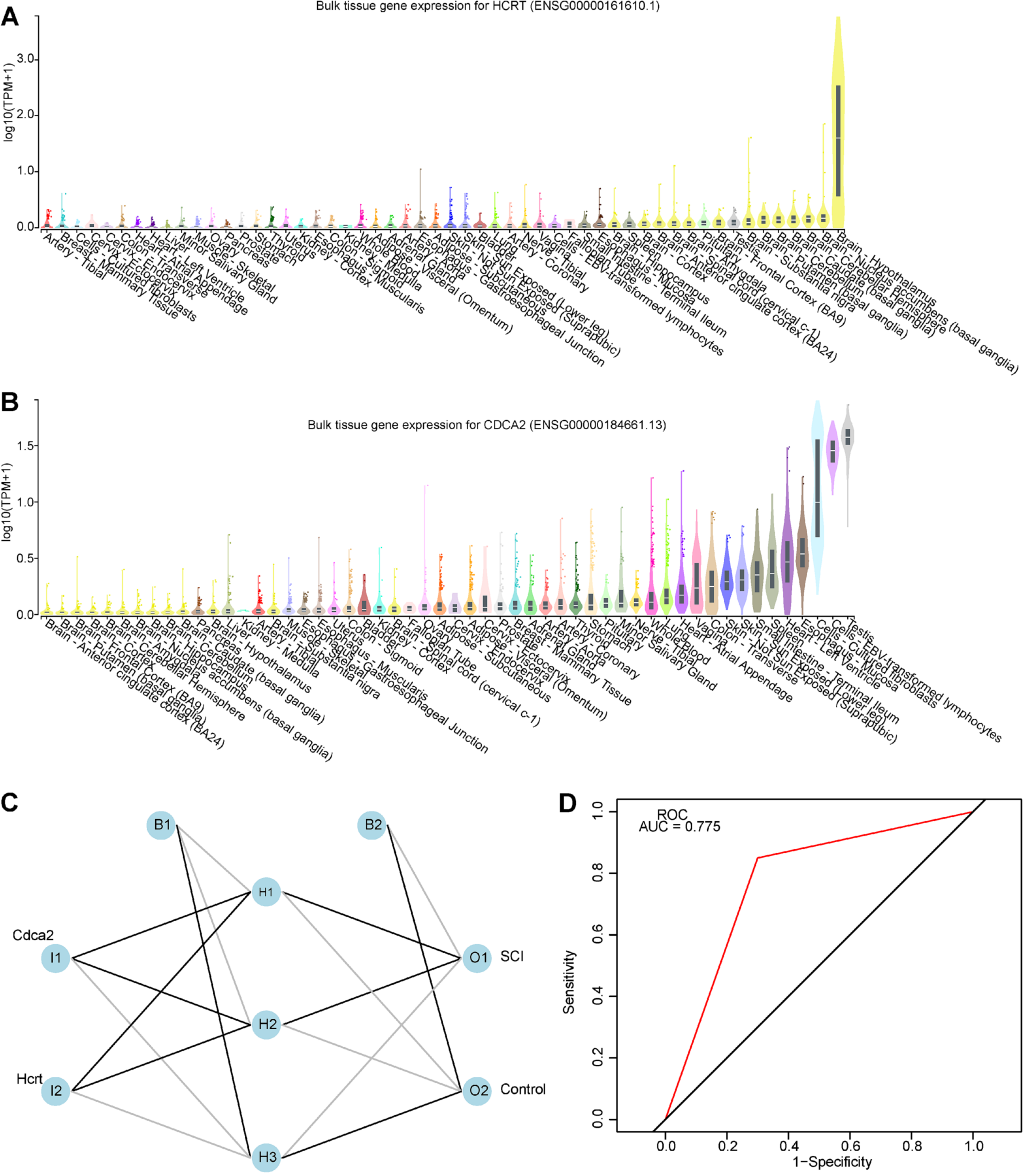
Tissue-specificity analysis and construction of artificial neural networks. **(A)** Tissue-specificity analysis network diagram for biomarker Hcrt, with the yellow portion of the diagram representing the brain-spinal cord region. **(B)** Tissue-specificity analysis network diagram for biomarker Cdca2, with the yellow portion of the diagram representing the brain-spinal cord region. **(C)** Artificial neural network model prediction. **(D)** The ROC curve of the artificial neural network model, with an AUC greater than 0.7, indicating a high prediction accuracy.

The GTEx database was utilized to assess the expression levels of the two biomarkers across various tissues under physiological conditions. Hcrt exhibited higher expression in the brain, particularly in regions such as the hypothalamus, nucleus accumbens, frontal cortex, amygdala, and spinal cord (Figure 4A). In contrast, Cdca2 was predominantly expressed in tissues like the testis, EBV-transformed lymphocytes, cultured fibroblasts, and esophageal mucosa (Figure 4B). Notably, Cdca2 was also detectable in the brain, with the highest expression observed in the spinal cord.

### Building an effective artificial neural network for diagnosing SCI

An ANN model was developed using the GSE109902 dataset to improve differentiation between SCI and control samples by integrating the expression of two biomarkers (Figure 4C). The ROC curve yielded an AUC value of 0.775, suggesting that the ANN model exhibited robust accuracy in predicting SCI (Figure 4D).

### Elucidating the biological mechanisms of biomarkers

**Figure 5:**
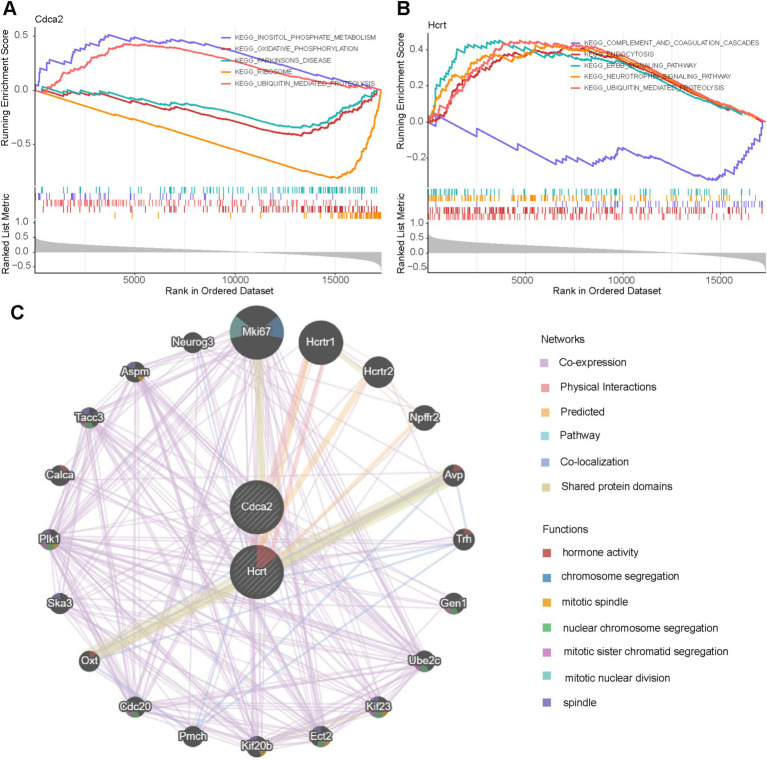
Signaling pathways and potential biological mechanisms involved by biomarkers and the construction of Gene MANIA networks. **(A,B)** GSEA enrichment analysis of biomarkers. **(C)** GeneMANIA network diagram.

The potential biological mechanisms of the biomarkers were analyzed using GSEA, identifying 19 enriched pathways for Hcrt and 16 for Cdca2 (adj. *p* < 0.05 and |NES| >1). Shared enriched pathways included “ubiquitin-mediated proteolysis,” “endocytosis,” and the “neurotrophin signaling pathway” (Supplementary Tables S3, S4). Cdca2 was also associated with pathways such as “oxidative phosphorylation,” “cytokine-cytokine receptor interaction,” “inositol phosphate metabolism,” and “sphingolipid metabolism” (Figure 5A). Hcrt expression showed significant correlations with the “ErbB signaling pathway,” “insulin signaling pathway,” “mTOR signaling pathway,” and “primary immunodeficiency,” among others. The top five pathways for each biomarker were visualized according to pathway significance (Figure 5B).

Additionally, co-expressed gene networks for the biomarkers were constructed using the GeneMANIA platform, comprising 39.02% co-expression, 25.61% physical interactions, 21.99% predicted interactions, 8.15% pathways, 2.86% co-localization, and 2.37% shared protein domains (Figure 5C). Both biomarkers were linked to 20 functionally related genes (e.g., Mki67, Hcrtr1, Hcrtr2), participating in biological processes such as “hormone activity,” “chromosome segregation,” and “mitotic spindle” organization.

### Biomarkers were linked to immune infiltrating cells in SCI

**Figure 6:**
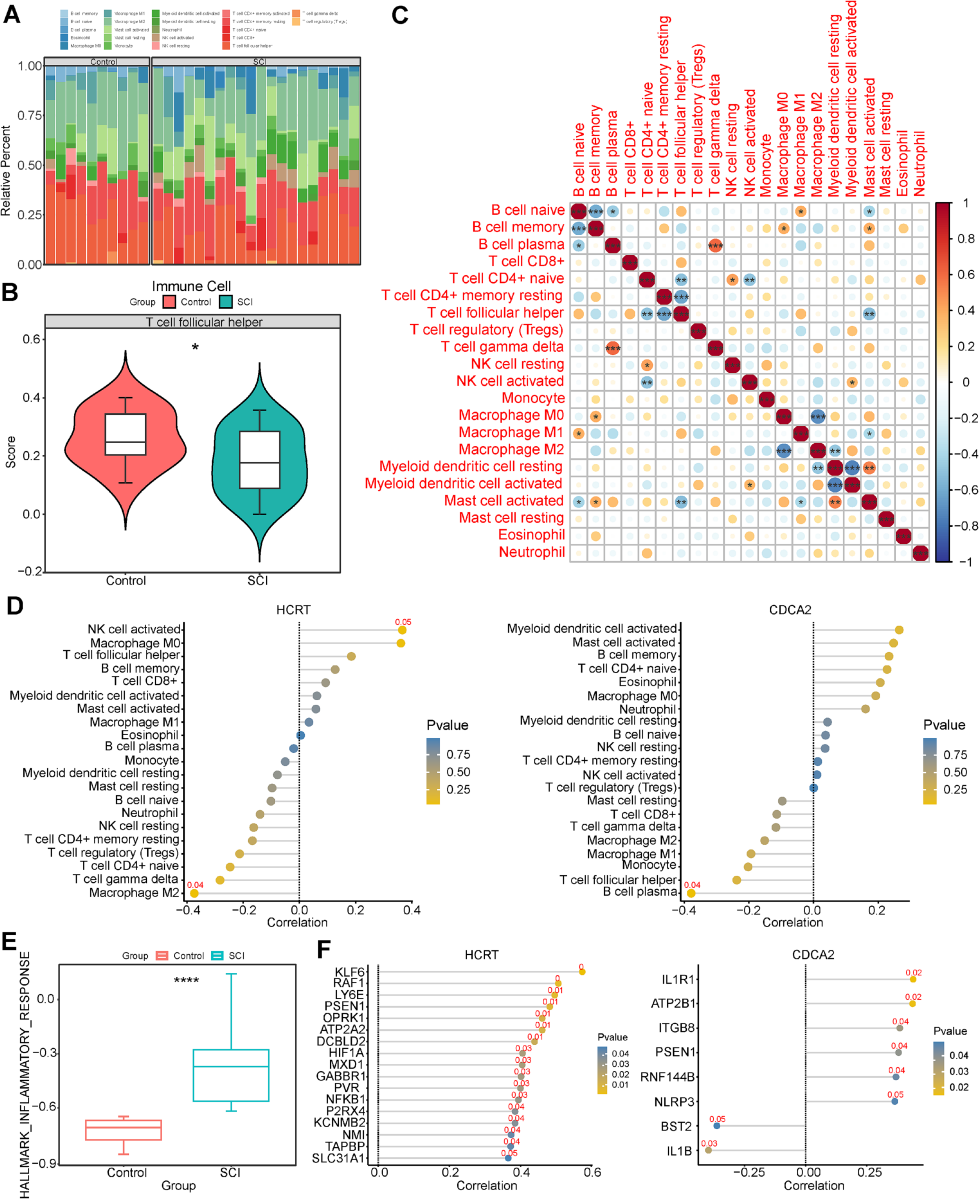
Immune infiltration analysis of biomarkers and analysis of correlation with inflammatory factors. **(A)** Immune cell infiltration between SCI and control groups. **(B)** Differences in immune cells between SCI and controls. **(C)** Correlation between immune cells. **(D)** Correlation between biomarkers and immune cells. **(E)** ssGSEA scores of inflammation-related genes in SCI samples and normal samples. **(F)** Correlation between biomarker and immune cells.

The infiltration levels of 22 immune cell types in each sample from the GSE109902 dataset were calculated using the CIBERSORT algorithm, as illustrated in Figure 6A. The Wilcoxon test revealed significant differences in T follicular helper cell levels between SCI and control samples, with lower levels observed in SCI samples (*p* < 0.05) (Figure 6B). Correlation analysis indicated that T follicular helper cells were negatively associated with naive CD4^+^ T cells (cor = −0.51, *p* = 0.004), activated mast cells (cor = −0.53, *p* = 0.003), and resting memory CD4^+^ T cells (cor = −0.63, *p* < 0.001) (Figure 6C). Biomarker-immune cell correlation analysis further demonstrated a positive association between T follicular helper cells and Hcrt, alongside a negative association with Cdca2 (Figure 6D).

### Biomarkers were linked to inflammatory factors in SCI

The inflammatory response plays a key role in the pathophysiology of SCI, with immune factors critically influencing its development and progression. The relationship between two specific biomarkers and 200 IRGs was assessed. Analysis using the ssGSEA algorithm revealed a significant difference in IRG scores between SCI and control samples in the GSE109902 dataset (*p* < 0.05), with higher scores detected in the SCI samples (Figure 6E). Correlation analysis showed that Hcrt exhibited significant positive correlations with 10 IRGs (e.g., KLF6, RAF1, LY6E), while Cdca2 demonstrated significant positive correlations with six IRGs (e.g., IL1R1, ATP2B1, PSEN1) and significant negative correlations with two IRGs (BST2 and IL1B) (|cor| >0.3, *p* < 0.05) (Figure 6F). Notably, both Hcrt and Cdca2 were strongly positively correlated with PSEN1.

### Potential regulatory mechanisms of biomarkers

**Figure 7:**
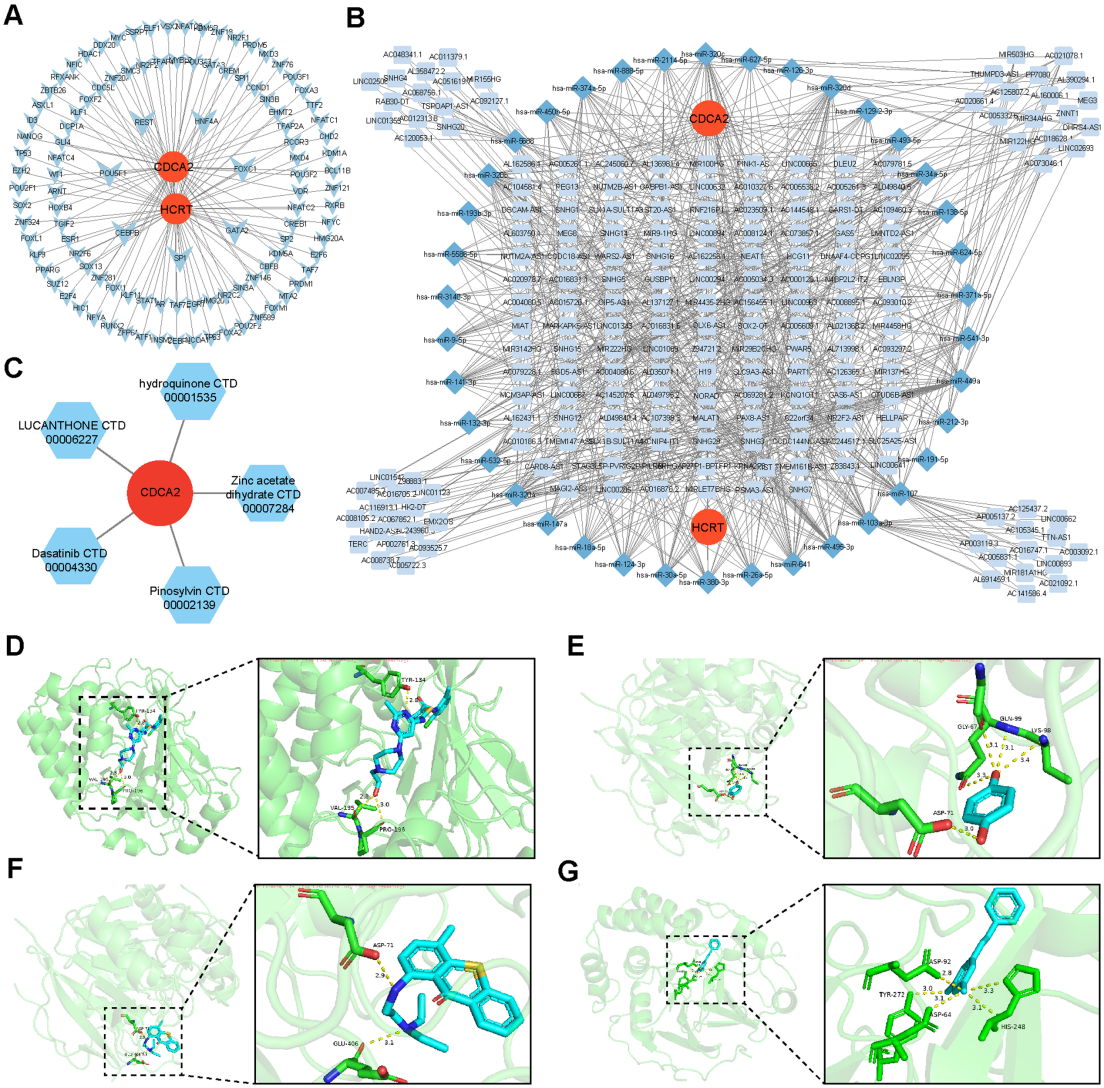
Molecular regulatory network of biomarkers and predicted targeted candidate drugs. **(A)** Biomarkers-TFs regulatory networks. **(B)** Biomarker-miRNA-lncRNA regulatory network. **(C)** Biomarker-drug association network diagram. **(D–G)** Molecular docking results.

Analysis of the miRNet database identified 110 TFs regulating the biomarkers, among which seven TFs (CEBPB, FOXC1, GATA2, HNF4A, REST, and SP1) were recognized as co-regulators of Hcrt and Cdca2 within the TF-biomarker network (Figure 7A). Additionally, predictions from individual databases identified 54 miRNAs targeting the biomarkers and 533 lncRNAs targeting these miRNAs. Within the lncRNAs-miRNAs-mRNAs network, numerous interaction pairs were observed (Figure 7B). For instance, lncRNAs such as MALAT1, NEAT1, and XIST were predicted to regulate Hcrt expression via hsa-miR-30a-5p, while MALAT1 and XIST were also linked to the regulation of Cdca2 expression through miRNAs like hsa-miR-124-3p and hsa-miR-132-3p. These interactions suggest potential therapeutic targets for SCI.

### Profiling drugs associated with biomarkers

Drug prediction via the Enrichr database identified five potential candidates associated with Cdca2: pinosylvin, zinc acetate dihydrate, hydroquinone, lucanthone, and dasatinib (Figure 7C). Notably, no drugs were predicted to interact with Hcrt. Molecular docking analysis demonstrated that all four drugs exhibited binding energies to Cdca2 below −5 kcal/mol (Table 2), suggesting stable interactions. The docking patterns of these four drugs with Cdca2 were subsequently visualized (Figures 7D–G).

**Table 2:** The binding energy between biomarkers and active ingredients.

Gene	Protein	PBD ID	Molecular name	Minimum binding free energy (kcal/mol)
CDCA2	Cell division cycle-associated protein 2	5INB	Pinosylvin	−6.6
CDCA2	Cell division cycle-associated protein 2	5INB	Hydroquinone	−5.0
CDCA2	Cell division cycle-associated protein 2	5INB	Lucanthone	−6.6
CDCA2	Cell division cycle-associated protein 2	5INB	Dasatinib	−8.1

## Discussion

SCI represents a severe neurological and pathological condition characterized by extensive motor, sensory, and autonomic impairments (1). Progress in its treatment remains constrained due to the complex interplay of its temporospatial pathological mechanisms, which are intricately connected and challenging to define with precision. The limited capacity for neural regeneration continues to present significant hurdles, with no effective strategies yet available to overcome this bottleneck (28). Secondary injuries following SCI are closely associated with mitochondrial dysfunction and ROS production (29). Ferroptosis, an iron-dependent cell death mechanism marked by lipid peroxidation and disruptions in iron metabolism, is critically influenced by mitochondrial ROS, which promote lipid peroxidation and may trigger ferroptosis (30). Despite these insights, the molecular mechanisms underlying mitochondrial dysfunction and ferroptosis in SCI remain poorly understood. To address this, the study identified two biomarkers, Hcrt and Cdca2, linked to mitochondrial dysfunction and ferroptosis, through transcriptomic analysis of SCI models combined with bioinformatics approaches. Additional analyses, including GSEA, immune infiltration, and drug prediction, were performed. These results offer valuable insights into the pathogenesis of SCI and provide a foundation for developing innovative therapeutic strategies to improve patient outcomes.

The Hcrt gene encodes orexin, also known as hypocretin, first identified in 1998 by Lecea at the Scripps Research Institute and Sakurai at Southwestern Medical Center in Texas cloned the gene expressed in the lateral hypothalamus of rats, identifying a neuropeptide structurally similar to secretin and naming it hypocretin (31). Neuropeptides, a class of peptides synthesized by neurons and neuroendocrine cells in the central nervous system, bind to specific surface receptors to regulate neuronal activity. Their role in SCI primarily involves influencing neuronal function and survival. In the context of SCI, neuropeptides modulate neuronal excitability, synaptic plasticity, and synchronized neural activity, thereby impacting functional recovery (32). Hcrt is hypothesized to regulate SCI recovery by modulating neuropeptide signaling pathways, offering novel perspectives and potential therapeutic strategies for SCI treatment.

Cdca2 functions as a regulatory factor in cell cycle progression and acts as a subunit of phosphatase 1γ (PP1γ), contributing to critical cellular processes such as chromosome segregation, nuclear envelope reconstruction, microtubule organization, and DNA damage repair (33). In the context of SCI, cell cycle regulation significantly influences post-injury repair and regeneration. Alterations in the cell cycle after SCI are closely associated with neuronal survival, regeneration, and glial cell responses (34). Neuronal regenerative capacity is inherently limited after SCI, largely because mature neurons typically remain in the G0 phase, restricting their ability to re-enter the cell cycle for division and repair. Additionally, glial scar formation, a major barrier to axonal regeneration, is linked to glial cell proliferation and cell cycle regulation (35). Further investigation is required to clarify the specific role of Cdca2 in modulating cell cycle dynamics following SCI. CDCA2 can activate the BRCA1-NRF2 signaling pathway (36). This cascade reaction enhances cellular antioxidant capacity and effectively reduces the content of reactive oxygen species, thus highlighting the complex role of CDCA2 in regulating cellular redox homeostasis. Its mechanism is not only reflected in direct participation in the production and removal of ROS but also in the activation of downstream antioxidant signaling pathways, thereby maintaining intracellular redox balance and potentially influencing mitochondrial function and ferroptosis processes, ultimately impacting SCI. To date, no research has been published investigating the roles of Hcrt and Cdca2 in spinal cord injury. The next step involves validating these findings across various species to deepen our understanding of this critical area of study.

GSEA enrichment analysis identified significant enrichment of Hcrt in the neurotrophin and ErbB signaling pathways, while Cdca2 was prominently associated with the oxidative phosphorylation and mTOR signaling pathways. The Hcrt gene, encoding orexin, is enriched in the ErbB signaling pathway, suggesting a potential role in the transformation of oligodendrocyte precursor cells and spontaneous remyelination following SCI, thereby contributing to its progression (37). The mTOR pathway, regulated by the mammalian target of rapamycin, is linked to the modulation of inflammation, apoptosis, and autophagy in SCI (38). Moreover, natural compounds derived from herbs and nutritional supplements may influence autophagy by targeting the mTOR pathway, presenting a potential therapeutic avenue in SCI management (39, 40). The association of Cdca2 with the oxidative phosphorylation pathway suggests its involvement in SCI progression through the regulation of mitochondrial respiration and oxidative phosphorylation (41). This analysis establishes a basis for exploring the roles of Hcrt and Cdca2 in SCI pathophysiology and highlights potential therapeutic interventions targeting these pathways.

The immunological analysis identified a significant reduction in T follicular helper cells (Tfh) in the SCI group compared to controls. SCI-induced immune deficiency syndrome (SCI-IDS), marked by systemic immunosuppression following SCI, substantially elevates the risk of infection and complicates therapeutic interventions. Research on the immune microenvironment emphasizes the role of Tfh cells in maintaining and refining immune system functionality. Evidence indicates that acute SCI downregulates CCR7 in peripheral tissues, reducing Tfh cell levels via chemokine signaling pathways, thereby contributing to SCI-IDS and worsening acute SCI (42). These results imply that Hcrt may influence immune responses through Tfh cells, thereby impacting SCI progression. Correlation analysis with inflammatory factors identified significant associations between the biomarkers and the inflammatory gene PSEN1. Mutations in PSEN1 have been implicated in atypical Alzheimer’s disease (AD) and non-AD phenotypes, including frontotemporal lobar degeneration (FTD), Parkinson’s disease (PD), dementia with Lewy bodies (DLB), and spastic paraplegia (SP) (43). PSEN1, a component of the γ-secretase complex, regulates APP cleavage and may also modulate processes such as Notch signaling, β-catenin processing, and calcium homeostasis (44). Further investigation is needed to clarify the role of PSEN1 in SCI and its potential as a mediator in the onset and progression of the condition.

An mRNA-miRNA-lncRNA network was constructed, identifying 54 miRNAs (e.g., miR-126, miR-132, miR-124) as potential regulators of the biomarkers, implicating their involvement in the post-transcriptional regulation of gene expression associated with SCI. Among them, miR-126 has been shown to promote angiogenesis and inhibit vascular inflammation in endothelial cells by targeting genes such as SPRED1, PIK3R2, and VCAM1, highlighting its regulatory role in vascular repair and inflammation following SCI (45). Additionally, miR-124 not only activates macrophages but also promotes their polarization from the M1 to the M2 phenotype, sustaining the M2 phenotype through enhanced miR-124 expression. This positions miR-124 as a key modulator of microglia/macrophage activity in the central nervous system. Evidence indicates that miR-124-3p, interacting with neuron-derived exosomes, suppresses M1 microglia activation via the MYH9/PI3K/AKT/NF-κB signaling pathway, contributing to improved functional recovery after SCI (46).

This study identified 533 lncRNAs, including MALAT1, NEAT1, and XIST. Evidence suggests that MALAT1 interacts with Nrf2 to suppress neuron apoptosis associated with SCI (47). Additionally, NEAT1 silencing has been shown to mitigate spinal cord injury and decrease cavity formation by upregulating miR-29b (48). Knockdown of NEAT1 also significantly attenuated SCI-related inflammation via the miR-211-5p/MAPK1 axis. Furthermore, the analysis revealed that lncRNAs such as MALAT1, NEAT1, and XIST regulated Hcrt expression through hsa-miR-30a-5p, while MALAT1 and XIST modulated Cdca2 expression via miRNAs such as hsa-miR-124-3p and hsa-miR-132-3p. Experimental validation remains necessary to substantiate these results.

Analysis using the Enrichr database identified five potential drugs interacting with Cdca2: pinosylvin, zinc acetate dihydrate, hydroquinone, lucanthone, and dasatinib. Molecular docking was performed to evaluate these interactions, with dasatinib demonstrating the lowest binding energy to Cdca2, indicative of the strongest intermolecular interaction. Dasatinib, an oral second-generation tyrosine kinase inhibitor, is widely used for treating Philadelphia chromosome-positive (Ph^+^) chronic myeloid leukemia (CML) and Ph^+^ acute lymphoblastic leukemia (ALL) (49). Beyond its established applications, dasatinib has been reported to modulate the LPS-induced neuroinflammatory response in microglia and astrocytes by inhibiting the AKT/STAT3 signaling pathway (50). Additionally, recent studies suggest that combining dasatinib with quercetin reverses senescence in LPS-stimulated primary cultured astrocytes and decreases pro-inflammatory cytokine levels. This combination has shown efficacy in alleviating spinal cord neuroinflammation and reducing hypersensitivity in a rat model of chronic constriction injury of the sciatic nerve (51).

Modulation of immune cell activity, including microglia and astrocytes, by dasatinib may contribute to mitigating neuroinflammatory responses, a key factor in the pathological progression of SCI. As a potential anti-inflammatory agent, the therapeutic potential of dasatinib for SCI and its association with mitochondrial dysfunction and ferroptosis warrant further investigation. This study primarily examines the role of ferroptosis during the acute and subacute phases, specifically on the 2nd and 7th days post-SCI. Future investigations should prioritize comprehensive evaluation of the pharmacological efficacy and safety profiles of these agents in SCI management, with particular emphasis on establishing methodological frameworks to assess their clinical viability as potential therapeutic candidates.

This study identifies Cdca2 and Hcrt as biomarkers linked to mitochondrial function and ferroptosis in SCI, utilizing transcriptomic data, machine learning algorithms, and qPCR validation. The expression patterns of these biomarkers align with the dataset results, with statistically significant differences, confirming their relevance in SCI pathology. These findings offer valuable reference points and potential therapeutic targets for SCI treatment. Notably, ferroptosis may initiate within the first 2 h post-SCI, highlighting the importance of investigating earlier time points to elucidate its induction mechanisms. Additionally, the differential sensitivity of neural cell types to ferroptosis and the expression patterns of DEGs within these cells remain unexplored. Further research should focus on cell-specific ferroptosis sensitivity and its implications to uncover the distinct contributions of various neural cell types in SCI progression.

## Data Availability

The datasets presented in this study can be found in online repositories. The names of the repository/repositories and accession number(s) can be found in the article/Supplementary material.

## Footnotes

^1^ http://www.ncbi.nlm.nih.gov/geo/

^2^ http://www.broadinstitute.org/mitocarta

^3^ http://genemania.org/

^4^ https://www.mirnet.ca/

^5^ https://ngdc.cncb.ac.cn/databasecommons/database/id/1310

^6^ https://ngdc.cncb.ac.cn/databasecommons/database/id/167

^7^ https://dianalab.e-ce.uth.gr/tarbasev9

^8^ https://maayanlab.cloud/Enrichr/

^9^ https://www.rcsb.org/

^10^ https://cadd.labshare.cn/cb-dock2/php/index.php
